# Supplementary material for: Development of a mouse model expressing a bifunctional glutathione-synthesizing enzyme to study glutathione limitation in vivo
Source: J Biol Chem. 2024 Jan 11;300(2):105645. doi: 10.1016/j.jbc.2024.105645 (PMC10869265; doi:10.1016/j.jbc.2024.105645)
Supplement: Supporting Figures [file mmc1.pdf]

Figure S1

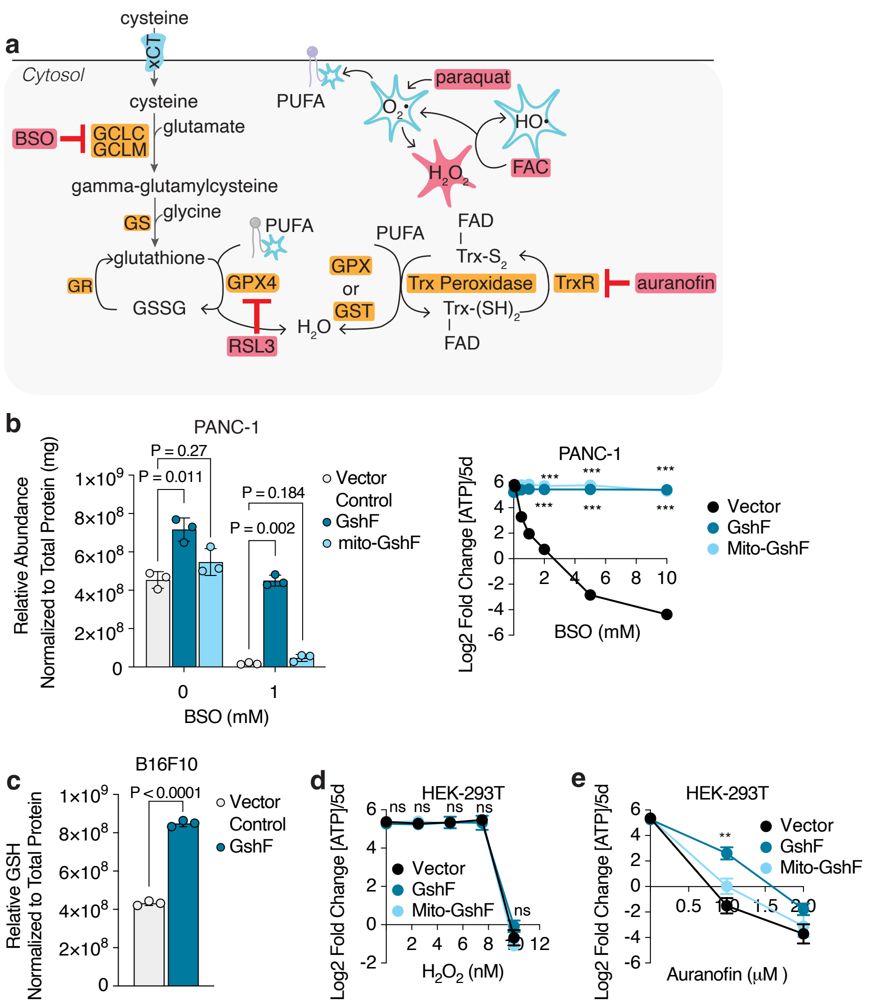

a
cysteine
xCT
Cytosol
cysteine
BSO
GCLC
GCLM
glutamate
gamma-glutamylcysteine
GS
glycine
PUFA
GR
glutathione
GSSG
GPX4
RSL3
H_2O
GPX
or
GST
PUFA
paraquat
O_2•
HO•
H_2O_2
FAC
FAD
$Trx-S_2$
Trx Peroxidase
$Trx-(SH)_2$
FAD
TrxR
auranofin
b
PANC-1
Relative Abundance
Normalized to Total Protein (mg)
P = 0.27
P = 0.011
P = 0.184
P = 0.002
Vector
Control
GshF
mito-GshF
BSO (mM)
PANC-1
Log2 Fold Change [ATP]/5d
BSO (mM)
Vector
GshF
Mito-GshF
c
B16F10
Relative GSH
Normalized to Total Protein
P < 0.0001
Vector
Control
GshF
d
HEK-293T
Log2 Fold Change [ATP]/5d
ns
Vector
GshF
Mito-GshF
H_2O_2 (nM)
e
HEK-293T
Log2 Fold Change [ATP]/5d
Vector
GshF
Mito-GshF
Auranofin (μM)

Figure S2

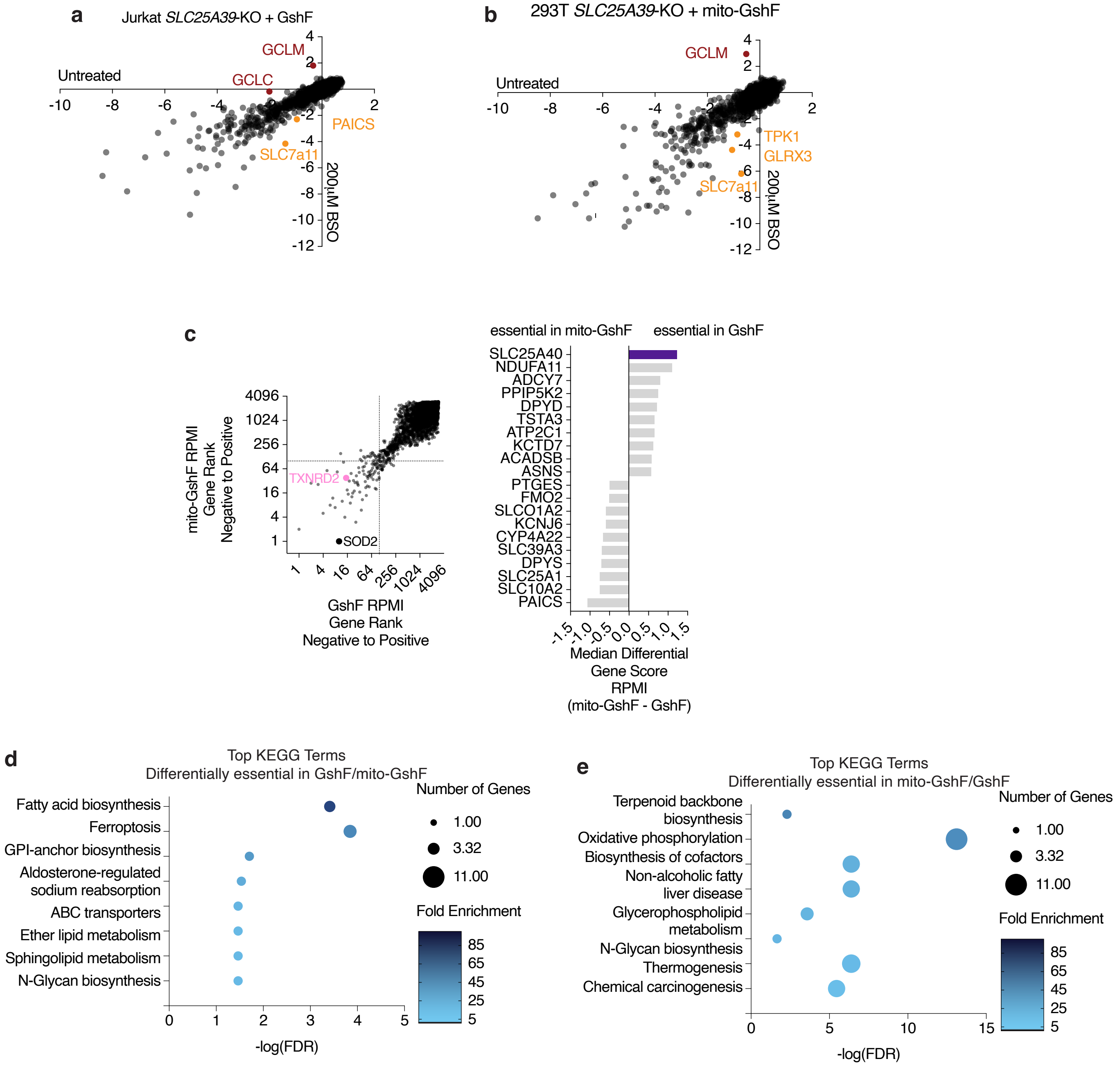
a
Jurkat SLC25A39-KO + GshF
Untreated
200μM BSO
GCLM
GCLC
PAICS
SLC7a11
-10
-8
-6
-4
-2
2
4
-12
b
293T SLC25A39-KO + mito-GshF
Untreated
200μM BSO
GCLM
TPK1
GLRX3
SLC7a11
c
mito-GshF RPMI
Gene Rank
Negative to Positive
GshF RPMI
Gene Rank
Negative to Positive
4096
1024
256
64
16
4
1
TXNRD2
SOD2
essential in mito-GshF
essential in GshF
SLC25A40
NDUFA11
ADCY7
PPIP5K2
DPYD
TSTA3
ATP2C1
KCTD7
ACADSB
ASNS
PTGES
FMO2
SLCO1A2
KCNJ6
CYP4A22
SLC39A3
DPYS
SLC25A1
SLC10A2
PAICS
-1.5
-1.0
-0.5
0.0
0.5
1.0
1.5
Median Differential
Gene Score
RPMI
(mito-GshF - GshF)
d
Top KEGG Terms
Differentially essential in GshF/mito-GshF
Fatty acid biosynthesis
Ferroptosis
GPI-anchor biosynthesis
Aldosterone-regulated
sodium reabsorption
ABC transporters
Ether lipid metabolism
Sphingolipid metabolism
N-Glycan biosynthesis
0
1
2
3
4
5
-log(FDR)
Number of Genes
1.00
3.32
11.00
Fold Enrichment
85
65
45
25
5
e
Top KEGG Terms
Differentially essential in mito-GshF/GshF
Terpenoid backbone
biosynthesis
Oxidative phosphorylation
Biosynthesis of cofactors
Non-alcoholic fatty
liver disease
Glycerophospholipid
metabolism
N-Glycan biosynthesis
Thermogenesis
Chemical carcinogenesis
0
5
10
15
-log(FDR)
Number of Genes
1.00
3.32
11.00
Fold Enrichment
85
65
45
25
5

Figure S3

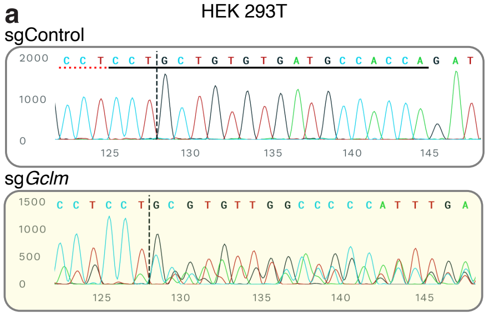

a
HEK 293T
sgControl
2000
1000
0
C C T C C T G C T G T G T G A T G C C A C C A G A T
125
130
135
140
145
sgGclm
1500
1000
500
0
C C T C C T G C G T G T T G G C C C C A T T T G A
125
130
135
140
145

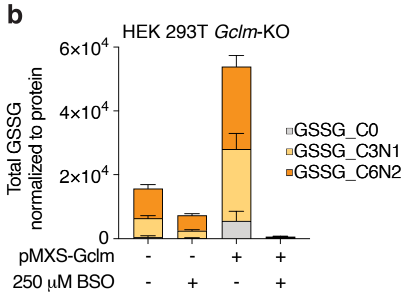

b
HEK 293T Gclm-KO
Total GSSG normalized to protein
6×10^4
4×10^4
2×10^4
0
GSSG_C0
GSSG_C3N1
GSSG_C6N2
pMXS-Gclm - - + +
250 μM BSO - + - +

Figure S4

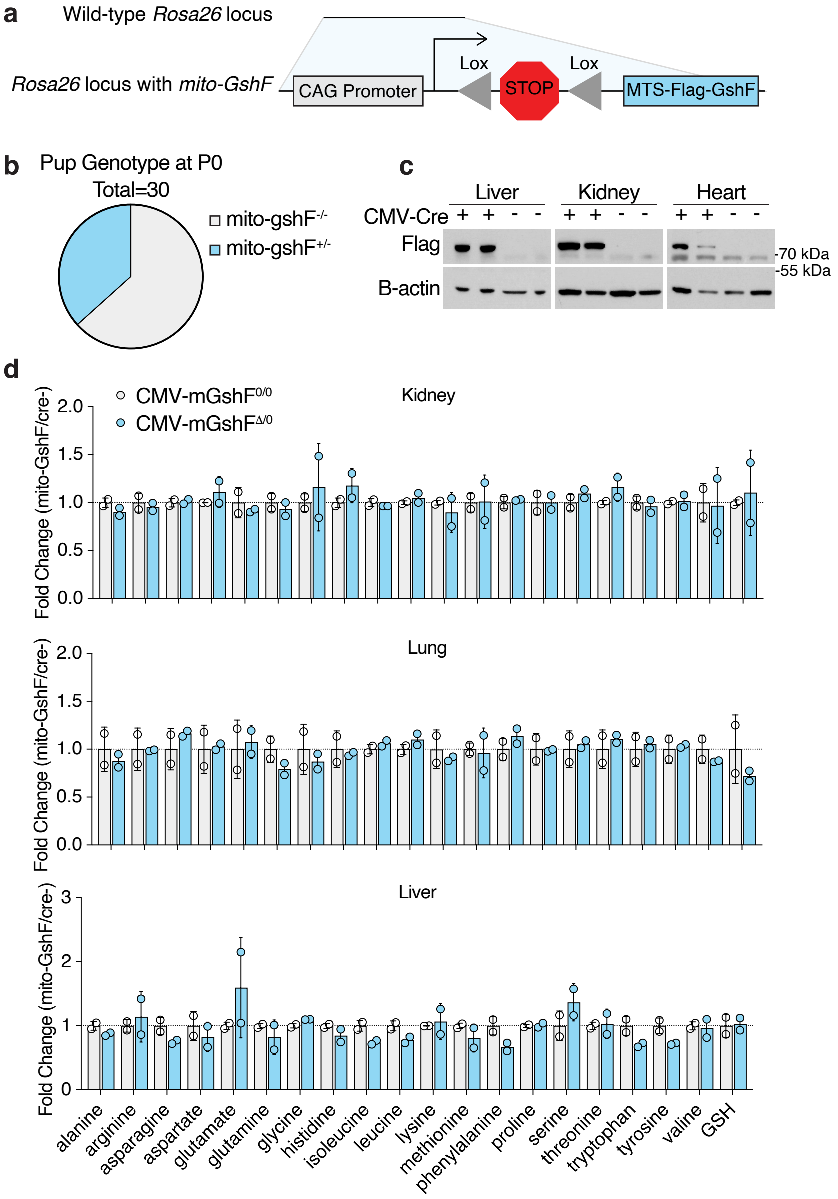

a
Wild-type Rosa26 locus
Rosa26 locus with mito-GshF
CAG Promoter
Lox
STOP
Lox
MTS-Flag-GshF
b
Pup Genotype at P0
Total=30
mito-gshF-/-
mito-gshF+/-
c
Liver
Kidney
Heart
CMV-Cre + + - -
Flag
B-actin
-70 kDa
-55 kDa
d
CMV-mGshF0/0
CMV-mGshFΔ/0
Kidney
Lung
Liver
Fold Change (mito-GshF/cre-)
alanine
arginine
asparagine
aspartate
glutamate
glutamine
glycine
histidine
isoleucine
leucine
lysine
methionine
phenylalanine
proline
serine
threonine
tryptophan
tyrosine
valine
GSH

Figure S5

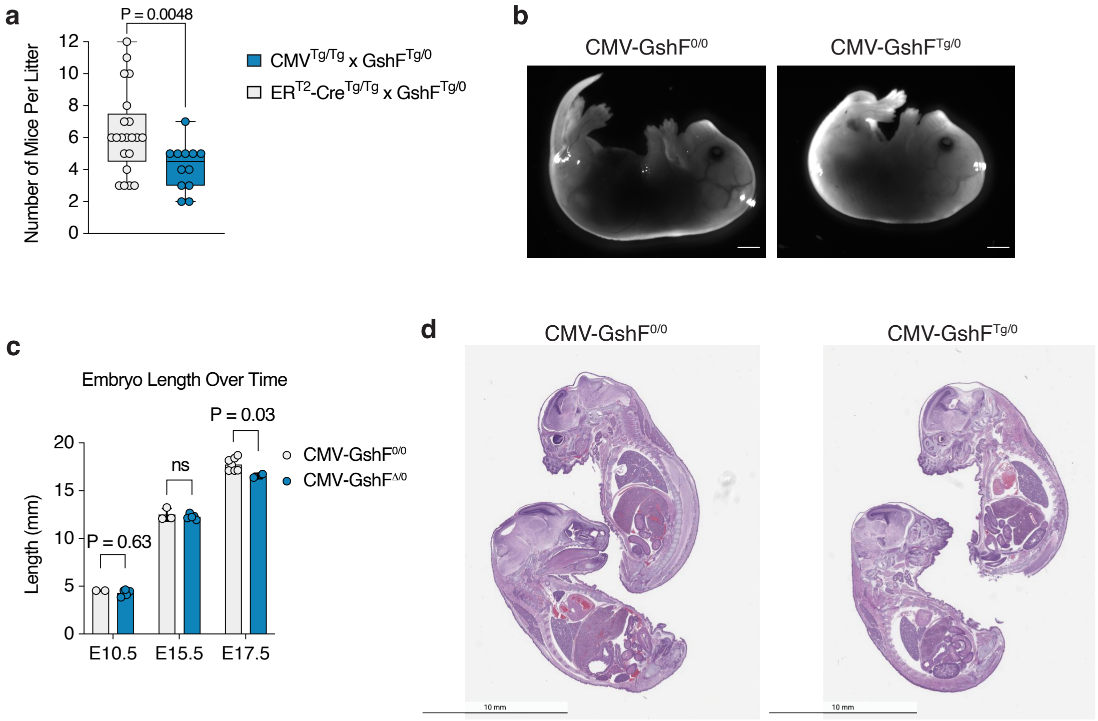
a
P = 0.0048
Number of Mice Per Litter
0
2
4
6
8
10
12
CMV$^{Tg/Tg}$ x GshF$^{Tg/0}$
ER^{T2}-Cre$^{Tg/Tg}$ x GshF$^{Tg/0}$
b
CMV-GshF$^{0/0}$
CMV-GshF$^{Tg/0}$
c
Embryo Length Over Time
Length (mm)
0
5
10
15
20
P = 0.63
ns
P = 0.03
E10.5
E15.5
E17.5
CMV-GshF$^{0/0}$
CMV-GshF$^{\Delta/0}$
d
CMV-GshF$^{0/0}$
CMV-GshF$^{Tg/0}$
10 mm
10 mm

Figure S6

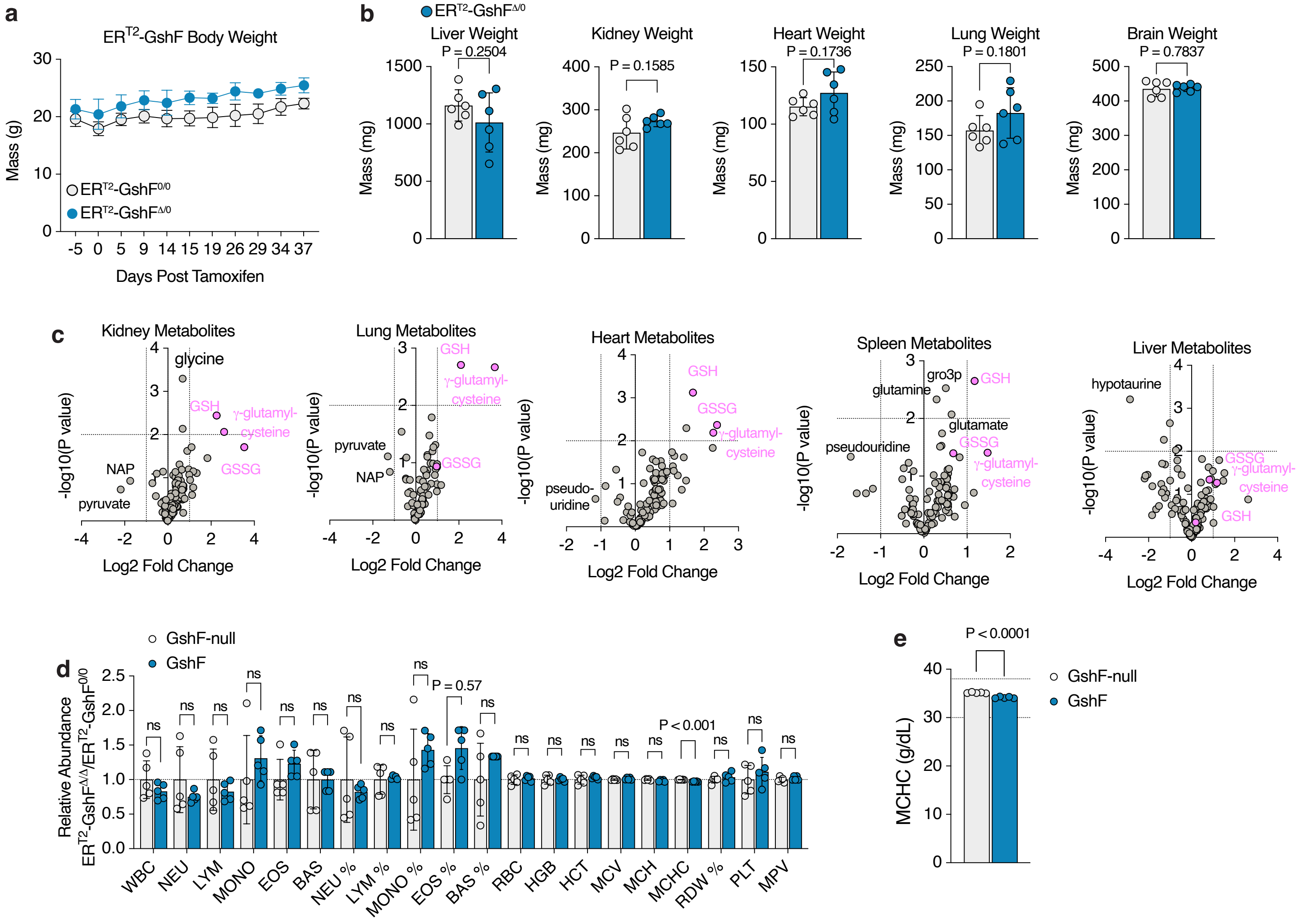
a
ERT2-GshF Body Weight
Mass (g)
Days Post Tamoxifen
ERT2-GshF0/0
ERT2-GshFΔ/0
b
Liver Weight
Kidney Weight
Heart Weight
Lung Weight
Brain Weight
P = 0.2504
P = 0.1585
P = 0.1736
P = 0.1801
P = 0.7837
Mass (mg)
c
Kidney Metabolites
Lung Metabolites
Heart Metabolites
Spleen Metabolites
Liver Metabolites
-log10(P value)
Log2 Fold Change
glycine
GSH
γ-glutamyl-cysteine
GSSG
NAP
pyruvate
pseudo-uridine
glutamine
gro3p
glutamate
pseudouridine
hypotaurine
d
GshF-null
GshF
Relative Abundance ERT2-GshFΔ/Δ/ERT2-GshF0/0
ns
P = 0.57
P < 0.001
WBC
NEU
LYM
MONO
EOS
BAS
NEU %
LYM %
MONO %
EOS %
BAS %
RBC
HGB
HCT
MCV
MCH
MCHC
RDW %
PLT
MPV
e
P < 0.0001
MCHC (g/dL)

Figure S7

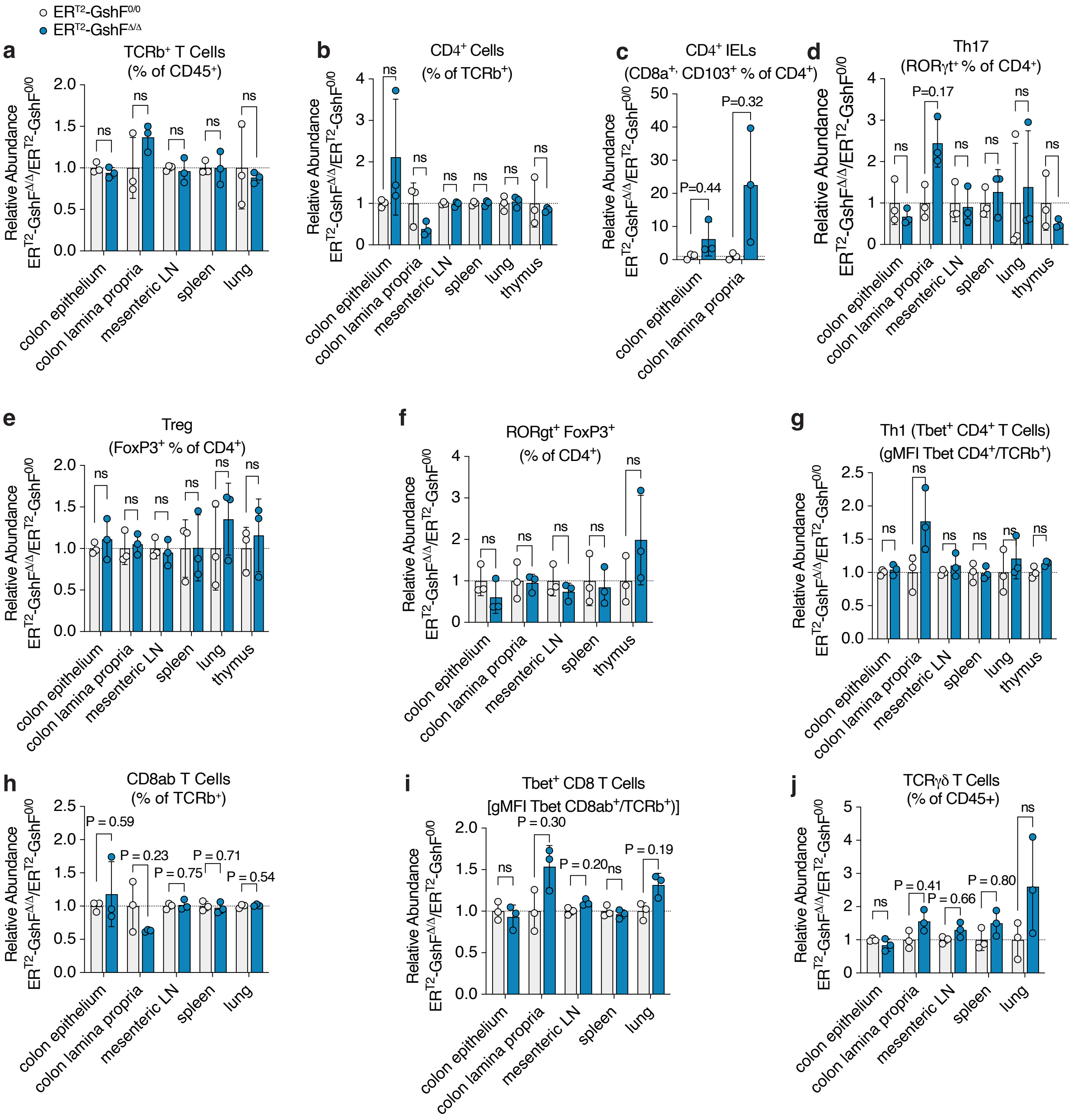

ERT2-GshF0/0
ERT2-GshFΔ/Δ
a
TCRb+ T Cells (% of CD45+)
b
CD4+ Cells (% of TCRb+)
c
CD4+ IELs (CD8a+, CD103+ % of CD4+)
P=0.44
P=0.32
d
Th17 (RORγt+ % of CD4+)
P=0.17
e
Treg (FoxP3+ % of CD4+)
f
RORgt+ FoxP3+ (% of CD4+)
g
Th1 (Tbet+ CD4+ T Cells) (gMFI Tbet CD4+/TCRb+)
h
CD8ab T Cells (% of TCRb+)
P = 0.59
P = 0.23
P = 0.75
P = 0.71
P = 0.54
i
Tbet+ CD8 T Cells [gMFI Tbet CD8ab+/TCRb+)]
P = 0.30
P = 0.20
P = 0.19
j
TCRγδ T Cells (% of CD45+)
P = 0.41
P = 0.66
P = 0.80
ns
Relative Abundance ERT2-GshFΔ/Δ/ERT2-GshF0/0
colon epithelium
colon lamina propria
mesenteric LN
spleen
lung
thymus
