# Supplementary material for: Development of a mouse model expressing a bifunctional glutathione-synthesizing enzyme to study glutathione limitation in vivo
Source: J Biol Chem. 2024 Jan 11;300(2):105645. doi: 10.1016/j.jbc.2024.105645 (PMC10869265; doi:10.1016/j.jbc.2024.105645)
Supplement: Supporting Information [file mmc2.docx]

**Supporting Figure Legends**

**Supporting Figure 1. GSH availability is not limiting for the oxidative stress response.**

1. Schematic of enzymatic inhibitors and ROS generators impacting glutathione metabolism and ferroptosis. BSO, buthionine sulfoximine; FAC, ferric (III) ammonium citrate.
2. Left, total abundance of GSH in mouse PANC-1 cells normalized by total protein (mg). Right, relative fold change in total ATP (log_2_) of PANC-1 cells transduced with *GshF*, *mito-GshF*, or a vector control and treated for 5 days with the indicated concentrations of BSO.
3. Total abundance of GSH in mouse B16F10 cells expressing indicated cDNAs and treated with vehicle or 1mM BSO for 72 hours. Data is normalized by total protein (mg).
4. Relative fold change in the total ATP (log_2_) of HEK-293T cells transduced with *GshF*, *mito-GshF*, or a vector control and treated for 5 days with the indicated concentrations of H_2_O_2_.
5. Relative fold change in the ATP (log_2_) of indicated cells transduced with *GshF*, *mito-GshF*, or a vector control and treated for 5 days with the indicated concentrations of auranofin. P < 0.05 are indicated by asterisks according to GraphPad style.

**Supporting Figure 2. A metabolism-targeted CRISPR-Cas9 screen reveals that unregulated GSH synthesis has little impact on most metabolic pathways.**

1. Left, gene scores of Jurkat *SLC25A39-*knockout cells expressing *GshF* or *mito-GshF* transduced with a CRISPR-Cas9 sgRNA library targeting 3,000 metabolic genes and grown in standard media for 14 doublings. The gene score is calculated as the median log_2_ fold change in all sgRNAs targeting a specific gene during the course of the culture period, as compared to a sample taken immediately before treatment began. Right, top 10 genes scoring as differentially required in cells expressing *GshF* compared to *mito-GshF* grown in normal culture conditions.
2. Gene ontology analysis of top 100 genes essential in Jurkat *SLC25A39*-knockout cells expressing *GshF* under BSO treatment.
3. Gene ontology analysis of top 100 genes essential in Jurkat *SLC25A39*-knockout cells expressing *mito-GshF* under BSO treatment.
4. Gene scores from Jurkat *SLC25A39*-knockout cells expressing *GshF* grown in standard medium compared to those treated with BSO for the course of the screen.
5. Gene scores from Jurkat *SLC25A39*-knockout cells expressing *mito-GshF* grown in standard medium compared to those treated with BSO for the course of the screen.

**Supporting Figure 3. Loss of the modifying subunit of the rate-limiting enzyme in GSH synthesis provides resistance to buthionine sulfoximine.**

1. Sanger sequencing of genomic region targeted by sg*Gclm* in HEK-293T cells transduced with indicated sgRNAs.
2. Total GSSG of indicated isotopes in the presence or absence of 250μM BSO in *Gclm*-knockout cells expressing indicated cDNAs.

**Supporting Figure 4. Constitutive mitochondrial glutathione synthesis is compatible with embryonic development.**

1. Schematic of *mito-GshF* insertion into the Rosa26 locus of CB57bl/6j mice.
2. Percent of CMV-mito-GshF ^Δ/0^ of the total litter. Expected ratio is 25%.
3. Immunoblots of mito-GshF expression in indicated tissues from CMV-mito-GshF ^Δ/0^ and CMV-mito-GshF ^0/0^ littermates.
4. Metabolomics analysis of indicated metabolites in CMV-mito-GshF ^Δ/0^ tissues, normalized to CMV-mito-GshF^0/0^ littermate controls.

**Supporting Figure 5. Constitutive unregulated glutathione synthesis is perinatal lethal.**

1. Litter size of indicated mating pairs.
2. Representative image of embryos of indicated genotypes at E17.5. Scale bar is 1mm.
3. Quantification of embryo length of respective genotypes at indicated stages of embryonic development.
4. Representative H&E stain of E17.5 mice of indicated genotypes. Scale bar represents 10mm.

**Supporting Figure 6. Unregulated glutathione synthesis in adult mice is compatible with life.**

1. Plot of body weights of littermates of indicated genotypes before and after tamoxifen treatment.
2. Weights of indicated tissues two weeks after tamoxifen induction.
3. Volcano plots of all metabolites detected in indicated tissues from male ER^T2^-GshF ^Δ/Δ^ mice compared to GshF ^fl/fl^ littermates.
4. Relative blood cell types or percentages from ER^T2^-GshF^Δ/Δ^ vs. ER^T2^-GshF^0/0^ littermates.
5. Relative mean corpuscular hemoglobin concentration (MCHC) cells from male ER^T2^-GshF^Δ/Δ^ vs. GshF^fl/fl^ littermates. Normal MCHC range is indicated by dotted lines.

**Supporting Figure 7. Mice with high GSH have normal immune profiles.**

1. Relative TCRb^+^ T cells in indicated tissues from female ER^T2^-GshF^Δ/Δ^ vs. ER^T2^-GshF^0/0^ littermates.
2. Relative CD4^+^ T cells in indicated tissues from female ER^T2^-GshF^Δ/Δ^ vs. ER^T2^-GshF^0/0^ littermates.
3. Relative CD4^+^ intraepithelial lymphocytes (IELs) cells in indicated colon layers from female ER^T2^-GshF^Δ/Δ^ vs. ER^T2^-GshF^0/0^ littermates.
4. Relative Th17 T cells in indicated tissues from female ER^T2^-GshF^Δ/Δ^ vs. ER^T2^-GshF^0/0^ littermates. P < 0.9 are indicated numerically.
5. Relative Treg cells in indicated tissues from female ER^T2^-GshF^Δ/Δ^ vs. ER^T2^-GshF^0/0^ littermates.
6. Relative RORγt^+^, FoxP3^+^, CD4^+^ T cells in indicated tissues from female ER^T2^-GshF^Δ/Δ^ vs. ER^T2^-GshF^0/0^ littermates.
7. Relative Th1 T cells in indicated tissues from female ER^T2^-GshF^Δ/Δ^ vs. ER^T2^-GshF^0/0^ littermates.
8. Relative CD8ab^+^ T cells in indicated tissues from female ER^T2^-GshF^Δ/Δ^ vs. ER^T2^-GshF^0/0^ littermates.
9. Relative CD8^+^ Tbet^+^ T cells in indicated tissues from female ER^T2^-GshF^Δ/Δ^ vs. ER^T2^-GshF^0/0^ littermates.
10. Relative TCRγδ T cells in indicated tissues from female ER^T2^-GshF^Δ/Δ^ vs. ER^T2^-GshF^0/0^ littermates.

**Supporting Table S1. A metabolism-targeted CRISPR-Cas9 screen reveals that unregulated GSH synthesis has little impact on most metabolic pathways.**

All gene scores from a metabolism-targeted CRISPR-Cas9 screen performed in Jurkat *SLC25A39*-knockout cells expressing *GshF* or *mito-GshF* and treated with or without 200μM BSO.

**Supporting Table S2. Differentially expressed genes in ERT2-GshF^Δ/Δ^ vs. GshF^0/0^ kidneys.**

All base means and log_2_ fold changes of mRNAs quantified during RNAseq from ERT2^Δ/Δ^ vs. GshF^0/0^ kidneys.

**Supporting Table S3. Differentially expressed genes in ERT2-GshF^Δ/Δ^ vs. GshF^0/0^ lungs.**

All base means and log_2_ fold changes of mRNAs quantified during RNAseq from ERT2^Δ/Δ^ vs. GshF^0/0^ lungs.
